# Extracellular Synthesis and Characterization of Silver Nanoparticles—Antibacterial Activity against Multidrug-Resistant Bacterial Strains

**DOI:** 10.3390/nano10020360

**Published:** 2020-02-19

**Authors:** Gajanan Ghodake, Min Kim, Jung-Suk Sung, Surendra Shinde, Jiwook Yang, Kyojung Hwang, Dae-Young Kim

**Affiliations:** 1 Department of Biological and Environmental Science, Dongguk University-Seoul, Biomedical Campus, 32 Dongguk-ro, Ilsandong-gu, Goyang-si 10326, Gyeonggi-do, Korea; ghodakegs@gmail.com (G.G.); shindesurendra9@gmail.com (S.S.); jwjii0@naver.com (J.Y.); kyojung7@naver.com (K.H.); 2 Department of Life Science, Dongguk University-Seoul, Biomedical Campus, 32 Dongguk-ro, Ilsandong-gu, Goyang-si 10326, Gyeonggi-do, Korea; pipikimmin@naver.com (M.K.); sungjs@dongguk.edu (J.-S.S.)

**Keywords:** extracellular nanosynthesis, green chemistry, silver nanoparticles, intracellular fluid discharge, pathogenic bacteria

## Abstract

Herein, we report the use of a cell-free extract for the extracellular synthesis of silver nanoparticles (AgNPs) and their potential to address the growing threat of multidrug-resistant (MDR) pathogenic bacteria. The reproducibility of AgNP synthesis was good and AgNP formation kinetics were monitored as a function of various reaction factors via ultraviolet-visible absorption spectroscopy. This green method was dependent on the alkaline pH of the reaction mixture. With the addition of dilute sodium hydroxide, well-dispersed AgNPs could be produced in large quantities via the classical nucleation and growth route. The new biosynthetic route enabled the production of AgNPs within a narrow size range of 4 to 17 nm. The AgNPs were characterized using various techniques and their antibacterial activity against MDR pathogenic bacteria was evaluated. Field-emission scanning electron microscopic imaging revealed prominent morphological changes in *Staphylococcus aureus* cells due to mechanical damage, which led to cell death. *Escherichia coli* cells showed signs of contraction and intracellular fluid discharge as a consequence of disrupted cell membrane function. This new biologically-assisted extracellular strategy is potentially useful for the decontamination of surfaces and is expected to contribute to the development of new products containing AgNPs.

## 1. Introduction

The frequent and widespread use of antibiotics in the last few decades has led to the development of antibiotic-resistant bacteria. In recent years, *Escherichia coli* (*E. coli*) and *Staphylococcus aureus* (*S. aureus*) have been identified as the sources for most common infections. Multidrug-resistant (MDR) pathogens are known to cause scalded skin syndrome, meningitis, endocarditis, osteomyelitis, food poisoning, urinary tract infections, pneumonia and diarrhea worldwide [1,2,3]. Resistance in pathogenic microorganisms poses a severe threat to global public health because conventional antibiotics will no longer be effective [4,5]. Thus, there is an urgent need for the development of novel antibiotics, antimicrobial agents and nanomaterials that exhibit strong antimicrobial activity without the induction of resistance in bacterial strains.

Oxide nanomaterials, including copper, zinc, cerium and iron, appear promising as antibacterial agents. However, oxide nanomaterials are limited by their poor dispersibility and stability in aqueous media [6,7,8]. Gold nanoparticles (NPs) are widely reported as antimicrobial agents in the scientific literature, despite their intrinsically non-antibacterial nature and the high cost involved in their manufacturing as well as commercial use [9]. In contrast, the intrinsic properties of silver (Ag) NPs make them promising antibacterial and bacteriostatic agents against a wide range of MDR strains [10]. The application of AgNPs in nanomedicine, antibacterial nanocomposites, disinfectants and antimicrobial coatings or wound healing products is projected to increase substantially in the next few decades [11,12].

Chemical and photochemical methods are commonly used for the synthesis of metal NPs as a colloidal dispersion in an organic solvent or aqueous solution by reducing their metal precursors. Such methods are usually costly, produce byproducts and hazardous waste and often require stabilizing agents. Additionally, extensive purification steps are necessary before the nanoproducts can be used in therapeutic or nanomedicine applications [13,14,15,16]. Most chemical methods also rely heavily on toxic chemicals and harsh organic solvents; this is typically due to the hydrophobic nature of the stabilizing agents used [17]. Therefore, there is a need to explore novel, cost-effective and green methods with scale-up capabilities for manufacturing AgNPs. With these considerations in mind, researchers need to investigate biological systems for bioinspiration [13,18].

The use of more sustainable and greener synthesis methods for producing metal NPs has many benefits from the perspective of green chemistry, including the choice of an aqueous medium, environmentally benign reducing agents and nontoxic solvents [17,19]. In general, the use of natural products, plant extracts, microbes or fungi as reducing agents reduces the requirement for chemical reducing agents such as hydrazine, sodium borohydride, ammonia or hydrogen gas, thus decreasing the waste byproducts and increasing the safety of the synthetic process and nanoproducts [14,20,21]. However, intracellular synthesis methods for metal NPs are hardly in favor due to losses in adsorption, cell disruption, purification steps, high costs and the potential to affect the physicochemical properties of metal NPs [22]. On the other hand, animal-source products (collagen, casein pancreatic digest, peptone, etc.) [23,24,25] are the most appropriate for extracellular production of metal NPs [26,27,28,29,30]. Thus, biosynthesis-based approaches would be attractive options if AgNP synthesis could be performed extracellularly using the extracted biomolecules present in cell-free beef extracts [29,31,32,33].

Herein, we report the use of cell-free extracts rich in free amino acids, organic acids and peptides [34] for the extracellular synthesis of colloidal AgNPs. The green method proposed is an attractive alternative to the aforementioned methods for metal NPs. This one-pot green method yields a concentrated suspension of colloidal AgNPs simply by reduction of the silver nitrate (AgNO_3_) precursor using an alkaline aqueous medium and a substantially low concentration of cell-free beef extract. The kinetics of AgNP formation were evaluated systematically and quantitatively using ultraviolet–visible (UV-vis) spectroscopy. AgNPs with the desired physicochemical properties, including size, shape and surface chemistry, could be obtained with high reproducibility. AgNP suspensions were used to evaluate their intrinsic antibacterial activity against MDR strains of *S. aureus* and *E. coli*. Field Emission Scanning Electron Microscopy (FE-SEM) and optical imaging results showed that the colloidal AgNPs could inhibit or stop the growth of pathogenic bacteria. The ease of synthesis, excellent storage stability, water solubility and potent bacteriostatic activity make AgNPs promising candidates for the manufacture of nanomedicines, disinfectants and surface treatment products.

## 2. Materials and Methods

### 2.1. Chemicals and Materials

AgNO_3_ was purchased from Sigma-Aldrich (St. Louis, MO, USA). Sodium hydroxide (NaOH) stock solution (1 M) was obtained from Daejung Chemicals (Daejung, Korea). Desiccated cell-free beef extract, nutrient broth (NB), Luria-Bertani (LB) medium and agar powder were purchased from Becton Dickinson (Pont-de-Claix, France). The Korean Culture Center of Microorganisms (KCCM, Seoul, Korea) supplied the MDR bacterial strains of *S. aureus* (KCCM 11335) and *E. coli* (KCCM 11234).

### 2.2. Extracellular Synthesis of AgNPs

**Figure 1:**
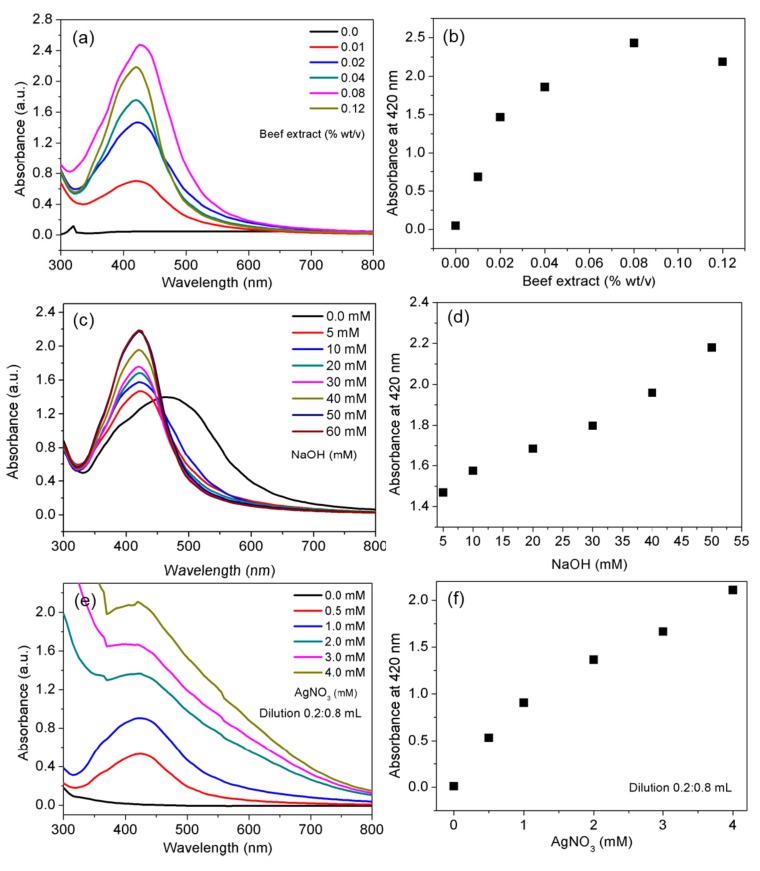
Effect of different parameters on the quality and yield of silver nanoparticles (AgNPs). UV-vis spectra of AgNPs prepared with various concentrations of cell-free beef extract (**a**); absorbance of AgNP suspensions prepared with various concentrations of cell-free beef extract at 420 nm (**b**); UV-vis spectra of AgNPs prepared with various concentrations of NaOH (**c**); absorbance of AgNP suspensions prepared with various concentrations of NaOH at 420 nm (**d**); UV-vis spectra of AgNPs prepared with various AgNO_3_ concentrations (**e**); and absorbance of AgNP suspensions prepared with various AgNO_3_ concentrations at 420 nm (**f**).

An aqueous solution of cell-free beef extract (0.4% *w*/*v*) was prepared in 50 mL nanopure water and mixed with gentle stirring for 2 min. The freshly prepared solution was stored in a refrigerator at 4 °C. The effect of the cell-free beef extract on AgNP production kinetics was evaluated by varying the concentration of the extract from 0.01% to 0.12% (*w*/*v*) at fixed AgNO_3_ (0.4 mM) and NaOH (20 mM) concentrations. The effect of NaOH was evaluated by preparing reaction mixtures with NaOH concentrations ranging from 5 to 60 mM at fixed AgNO_3_ (0.4 mM) and cell-free beef extract (0.08% *w*/*v*) concentrations. The effect of the initial AgNO_3_ concentration was evaluated from 0.5 to 4 mM at fixed concentrations of NaOH (20 mM) and cell-free beef extract (0.08% *w*/*v*). To initiate AgNP synthesis, the desired molar ratios of AgNO_3_ were added to the reaction mixtures after heating at 60 °C. The reactions were allowed to proceed for approximately 12 h at 60 °C. The completion of the reaction was indicated by a color change, with the solution turning dark yellow or brown. Aliquots of the AgNPs were collected from the reaction mixtures prepared with varying concentrations of AgNO_3_, diluted with nanopure water as shown in Figure 1. UV-vis absorbance spectra were collected using an Optizen 2120 spectrophotometer (Mecasys, Korea).

### 2.3. Characterization of AgNPs

The composition and chemical state of the AgNPs were investigated using X-ray photoelectron spectroscopy (XPS). The binding energies and areas of the Ag 3d, C 1s and O 1s photoelectron peaks were then analyzed. X-ray powder diffraction (XRD) analysis of the AgNP suspensions dried on a glass substrate was conducted using a PANalytical X’Pert Pro diffractometer (Malvern Panalytical, UK) equipped with a CuKα radiation source (λ = 1.54059 Å) at a scan speed of 2°/min. Fourier-transform infrared (FTIR) spectra of the AgNPs were obtained from 500 to 4,000 cm^−1^ using a Thermo Fisher Scientific (Nicolet iS10, Waltham, MA, USA) with a diamond attenuated total reflectance attachment. High-resolution transmission electron microscopy (HR-TEM) images and selected area electron diffraction (SAED) patterns were obtained using HF-3300 field-emission TEM (Hitachi, Japan). Samples for HR-TEM analysis were prepared by placing drops of the dilute AgNP suspensions on Formvar-coated copper grids.

### 2.4. Antimicrobial Susceptibility Assay: Minimum Inhibitory Concentration (MIC) and Minimum Bactericidal Concentration (MBC)

*S. aureus* and *E. coli* cells were harvested at the early stationary phase of growth and the cell concentration was adjusted to 10^6^ colony-forming units (CFUs)/mL. A turbidity assay was performed to determine the MIC value for the AgNPs. In brief, increasing concentrations of AgNPs (10–50 μg/mL) were mixed into sterile LB and NB media and inoculated with freshly cultured *S. aureus* and *E. coli* separately. These samples were incubated under shaking (100 rpm) at 37 °C and the absorbance intensity of the media sample was recorded using a UV-vis spectrophotometer at 600 nm for 36 h at different time intervals. The negative control experiment was performed using the cell-free beef extract. The bactericidal activity of the AgNPs was also assessed using the spread plate method. For MBC measurements, the surfaces of nutrient agar plates were inoculated for 24 h at 37 °C with bacterial cultures with or without the addition of AgNP suspensions ranging from 0 to 100 µg/mL.

### 2.5. Optical Microscopy

AgNP suspensions (30 µg/mL) were treated for 2 h with *E. coli* and *S. aureus* cells. The suspensions were then gently centrifuged at ~1,500 g for 15 min and the pellets were washed in phosphate-buffered saline (PBS, (1X, pH 7.4). The washed bacterial samples were resuspended in PBS and used for Gram staining. The bacterial suspensions in PBS were loaded onto slides and covered with glass coverslips. The bacterial cells were immobilized via gentle heat fixation, dried on the slides and then rehydrated with nanopure water. After mounting a slide onto the microscope, a 0.020 mL aliquot of 1% crystal violet (CV, Sigma-Aldrich, St. Louis, MO, USA) reagent was placed at the edge of the coverslip and suffused with Gram′s iodine solution (Sigma-Aldrich, St. Louis, MO, USA). The CV-iodine complexes were then counterstained with safranin [35] and used for the acquisition of Gram-stain images. Gram-stain image analysis of the sizes, shapes and morphologies of the bacteria was performed in triplicate.

### 2.6. Field-Emission Scanning Electron Microscopy

Coverslips were coated with poly-L-lysine (Sigma-Aldrich, St. Louis, MO, USA) to fix the bacterial cells following the protocol suggested by the manufacturer. Prior to the treatment with AgNPs, bacterial suspensions were mounted onto the poly-L-lysine-coated coverslips. The coverslips were soaked in PBS (Bio-sensing, Daejeon, Korea) at 37 °C and treated with a 30 µg/mL AgNP suspension for 2 h. After AgNP treatment, the bacterial cells were fixed in 2.5% glutaraldehyde for 24 h at 4 °C. The treated samples were sequentially dehydrated in 30%, 50%, 60%, 70%, 80%, 90% and 100% ethanol for 10 min. The fixed and treated samples were sputter-coated with platinum and the effect of the AgNPs on the morphological features of the bacteria was examined using S-4700 Field Emission Scanning Electron Microscope (FE-SEM, Hitachi S-4700, Tokyo, Japan).

## 3. Results and Discussion

### 3.1. Effect of Cell-Free Beef Extract Concentration

The effect of the cell-free beef extract was first tested at a fixed ratio of AgNO_3_ to NaOH (0.4:20 mM) for 12 h at 60 °C. AgNPs were prepared in cell-free beef extract solutions ranging in concentration from 0.01% to 0.12% (*w*/*v*). The UV-vis spectra for each dark yellow suspension contained a characteristic surface plasmon resonance (SPR) band at 420 nm (Figure 1a). The narrow SPR peaks indicated that the synthesis followed the classical nucleation and growth route. The results demonstrated that it was possible to obtain dispersed AgNPs, even with a low concentration of cell-free beef extract (0.01% *w*/*v*). The UV-vis absorption spectra and color of the suspensions remained characteristically identical as the concentration of the cell-free beef extract increased (Figure 1a), indicating continuous production of the desired AgNPs. As the concentration of the cell-free beef extract increased, the intensity of the SPR peak also increased. This suggested a quantitative increase in AgNP production (Figure 1b), in agreement with previously reported results [36]. It is noteworthy that the highest AgNP yield of ~97% was obtained at a cell-free beef extract concentration of 0.08% (*w*/*v*) based on the experimental absorbance intensity of the suspension. The absorption spectrum of dispersed AgNPs contained a single SPR band in the range of 405 to 420 nm. In contrast, published spectra for aggregated metal NPs contain either a broad SPR band or multiple peaks [37].

### 3.2. Effect of NaOH Concentration

The shape of the UV-vis spectra and AgNP formation depended on the NaOH concentration. The UV-vis spectra for the AgNPs were recorded after the reaction had progressed for 12 h (Figure 1c). The results indicated that the reactivity of the Ag complexes with the reducing agent increased with increasing NaOH concentration. A broad, low-intensity SPR band appeared in the spectrum when no NaOH was present in the reaction mixture (Figure 1c). A sharp SPR band appeared following NaOH (5 mM) addition, which was a visible indicator of AgNO_3_ reduction. A similar observation was made by Roopan et al. (2013), who reported that alkaline pH plays an important role in the production of dispersed NPs [38]. The intensity of the SPR band at 420 nm was measured at NaOH concentrations ranging from 5 to 60 mM. The UV-vis spectra showed a linear increase in absorbance intensity at 420 nm (Figure 1d). The strong dependence of Ag complex reduction on pH was evident, with cell-free beef extract acting as a reduction and stabilization agent. The alkaline pH of the reaction mixture had a crucial influence over the reaction rate, yield and stability of the AgNPs. Thus, the synergistic role of the biomolecules and the pH-dependent deprotonation of the functional groups could be precisely tuned by adding dilute NaOH to the reaction mixture. The UV-vis absorbance of AgNPs synthesized using olive leaf extract has been reported to increase with an increase in the pH of the reaction mixture [39].

### 3.3. Effect of AgNO_3_ Concentration

The production of AgNPs after AgNO_3_ reduction at 60 °C was monitored over an AgNO_3_ concentration range of 0.5 to 4.0 mM (Figure 1e,f). Each reaction mixture (0.2 mL) was diluted with 0.8 mL nanopure water prior to recording the UV-vis spectrum. The spectra are shown in Figure 1e. As the AgNO_3_ concentration in the reaction mixtures increased, the intensity of the SPR band increased linearly, with no shifts in the band. The absorption maxima appeared consistently at 420 nm at all tested AgNO_3_ concentrations, indicating the formation of dispersed AgNPs in high concentrations.

The absorbance intensity at 420 nm in Figure 1f was a function of the silver precursor concentration, even after the nanoproduct (0.2 mL) was diluted with 0.8 mL nanopure water. It is worth noting that a linear increase in the AgNP absorbance intensity was observed with no detrimental effects in the quality of the AgNPs up to an AgNO_3_ concentration of 4.0 mM. It was evident that as the concentration of Ag^+^ ions increased, the absorbance intensity increased, which reflected the increasing AgNP concentrations in the aqueous suspensions. The cell-free extract method was robust in terms of controlling the formation kinetics of AgNPs with the desired physicochemical properties. An excellent yield was successfully achieved with the use of a small amount of cell-free beef extract (0.08% *w*/*v*). This suggests that the method reduces or eliminates the need for extensive purification steps [19]. The method also adheres to the excellent “atom economy” principle of green chemistry. The cell-free beef extract served the dual purposes of reducing Ag complexes and stabilizing concentrated AgNP suspensions as previously reported [40].

### 3.4. Characterization of AgNPs

**Figure 2:**
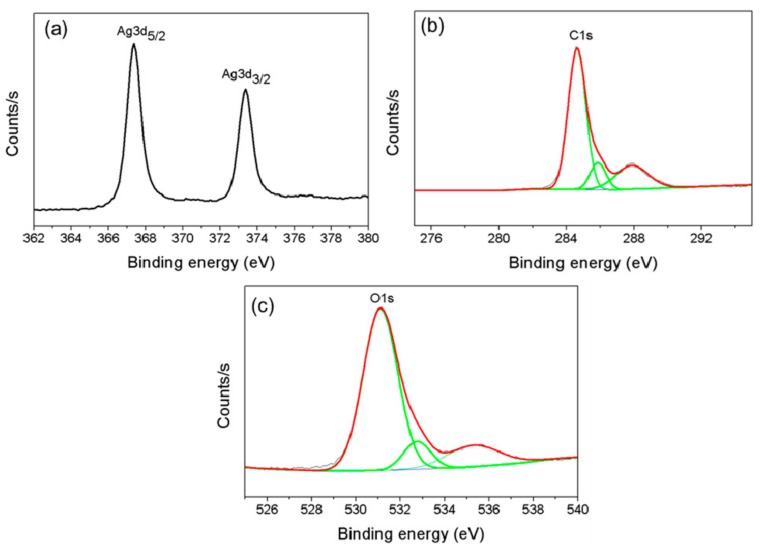
X-ray photoelectron spectra (XPS) of the AgNPs. Ag 3d spectrum (**a**), C 1s spectrum (**b**) and O 1s spectrum (**c**).

Precise control over the size distribution of NPs and their yield is a primary goal of nanoscience. Characterizing the physicochemical properties of metal NPs is important during the design and synthesis of NPs with the desired properties for target biomedical applications. AgNPs synthesized using 0.4 mM AgNO_3_, 0.08% (*w*/*v*) cell-free beef extract and 25 mM NaOH at 60 °C were used for XPS analysis, XRD, FTIR spectroscopy and HR-TEM imaging. XPS measurements were performed to obtain information about the chemical composition of the prepared AgNPs. The survey XPS spectrum in Figure 2 contains Ag 3d peaks, as well as C 1s and O 1s peaks that originated from C and O atoms in the biomolecules on the AgNP surfaces. The XPS data and 3d core levels of Ag in the AgNP thin films are shown in Figure 2a. The Ag 3d peaks centered at 367.4 eV and 373.5 eV with a spin-orbit separation of 6.1 eV were assigned to the Ag 3d_5/2_ and Ag 3d_3/2_ lines, respectively. Both values were consistent with pure metallic Ag [41]. The binding energy (BE) of 284.9 eV in the AgNP XPS spectrum in Figure 2c was referenced to the C 1s core level (Figure 2b). The peak at 531.1 eV in the high-resolution O 1 s spectrum (Figure 2c) was closely associated with the oxygen in carboxyl groups (C=O). The other peak at 535.2 eV was attributed to the oxygen in hydroxyl groups (−OH).

**Figure 3:**
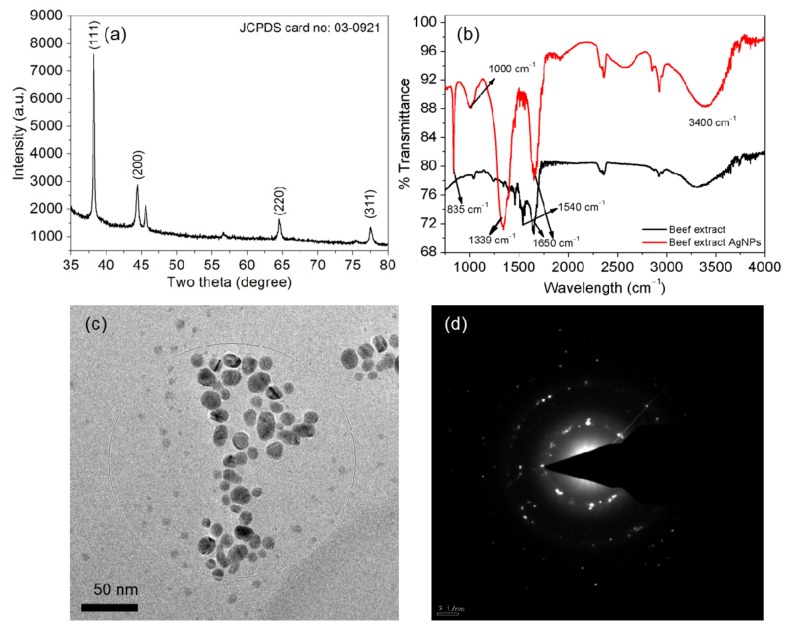
Characterization of the AgNPs. X-ray diffraction (XRD) pattern (**a**), Fourier transform infrared (FTIR) spectra (**b**), High-resolution transmission electron microscopy (HR-TEM) images (**c**) and AgNP selected area electron diffraction (SAED) pattern (**d**).

XRD analysis was conducted to identify the phases in the AgNPs and the crystallite size. Sharp peaks in the AgNP pattern at (2*θ*) 38.22°, 44.49°, 64.54° and 77.59° corresponded to the (111), (200), (220) and (311) planes of cubic Ag, respectively (Figure 3a). The FTIR spectrum for the AgNPs is presented in Figure 3b. The FTIR spectrum for the cell-free beef extract is included for reference (black line). The reference spectrum contained two main bands at 1650 and 1540 cm^–1^, which were assigned to the C=O stretching vibration of amide I and the C−N stretching vibration of amide II, respectively [42]. Fingerprint peaks assigned to amino acids or peptides appeared at 3400, 1650, 1540, 1339, 1000 and 835 cm^–1^. The prominent band in the region from 1200 to 1350 cm^–1^ originated from a combination of the in-phase C−N stretching and N−H bending vibrations of amide III. The FTIR spectra of the cell-free extract and the AgNPs contained an amide I band at approximately 1650 cm^–1^. However, the amide III bands were more prominent owing to significant capping by the peptides in the cell-free extract. The FTIR bands near 3,400 cm^–1^ were due to O–H stretching vibrations in amino acid residues in the cell-free extract. The broad FTIR band located at 1000 cm^−1^ was indicative of multiple interactions between the AgNPs and amino acid residues in the cell-free extract [43].

HR-TEM was used for structural characterization to evaluate the size, shape and size distribution of the AgNPs. Most of the synthesized AgNPs were passivated and had a narrow size distribution. The HR-TEM images revealed AgNPs with spherical or oval shapes and there was no sign of aggregation (Figure 3c). The size distribution was calculated from multiple images and the AgNPs ranged in size from 4 to 17 nm. Prominent ultra-small AgNPs approximately 4–6 nm in size were also visible. The SAED pattern for the AgNPs in the HR-TEM image is shown in Figure 3d. The SAED pattern showed clear diffraction rings with bright spots, which corresponded to the (111), (200), (220) and (311) crystallographic planes of AgNPs in a face-centered cubic lattice. The SAED pattern revealed crystalline AgNPs, which was in agreement with the results of XRD analysis and those in a previous report [44].

### 3.5. Stability Studies

**Figure 4:**
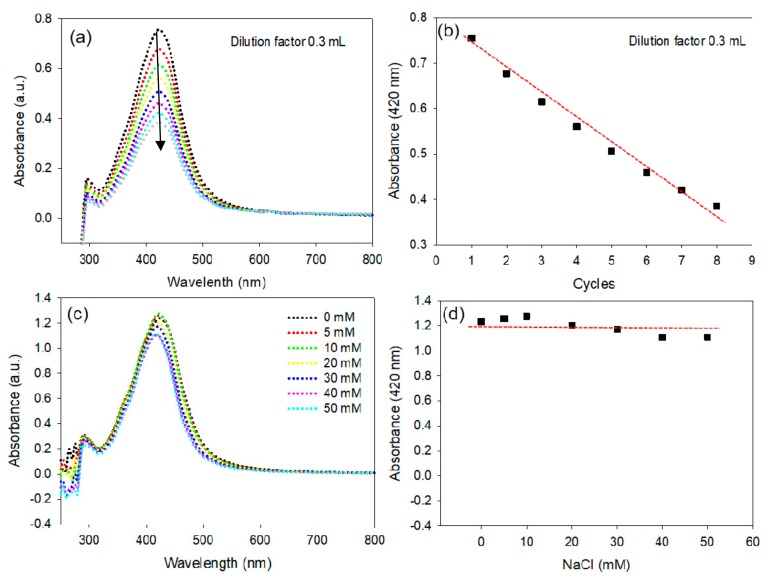
Stability of the AgNPs. Change in the UV-vis spectrum of AgNPs under various dilution conditions (**a**), Change in the plasmon absorption of AgNPs under a series of dilution conditions; experimental data; linear fit (**b**). Change in the UV-vis spectrum of AgNPs under increasing concentrations of NaCl (**c**), Change in the plasmon absorption of AgNPs under increasing concentrations of NaCl (**d**).

Employing AgNPs in biomedical applications requires low concentrations and it is vital to study the effects of dilution on AgNP solutions, as suggested in a previous report on AuNPs [45]. A detailed investigation was performed to determine the effects of dilution on the stability of AgNPs. The UV-vis spectra, bandwidth (Δλ) and plasmon resonance wavelength (λ_max_) were monitored after every consecutive addition of 0.3 mL of nanopure water to 1 mL of AgNP solution (Figure 4a). The absorption intensity at λmax (420 nm) was found to be linearly dependent on the dilution of AgNPs, in agreement with the Beer-Lambert law, as shown in Figure 4b. It is significant to note that the Δλ and λmax of the AgNPs did not change after a series of dilution cycles.

The stability of the AgNPs under increasing concentrations of NaCl was further evaluated to determine the effect of the ionic strength and robustness of the NPs. The data shown in Figure 4c,d demonstrates that the AgNPs have exceptional *in-vitro* stability, with the exception of a minor effect on the intensity of the λmax. The overall results suggest that the dilution of AgNPs and ionic strength up to 50 mM do not alter their characteristic physiochemical properties.

### 3.6. Antibacterial Results

**Figure 5:**
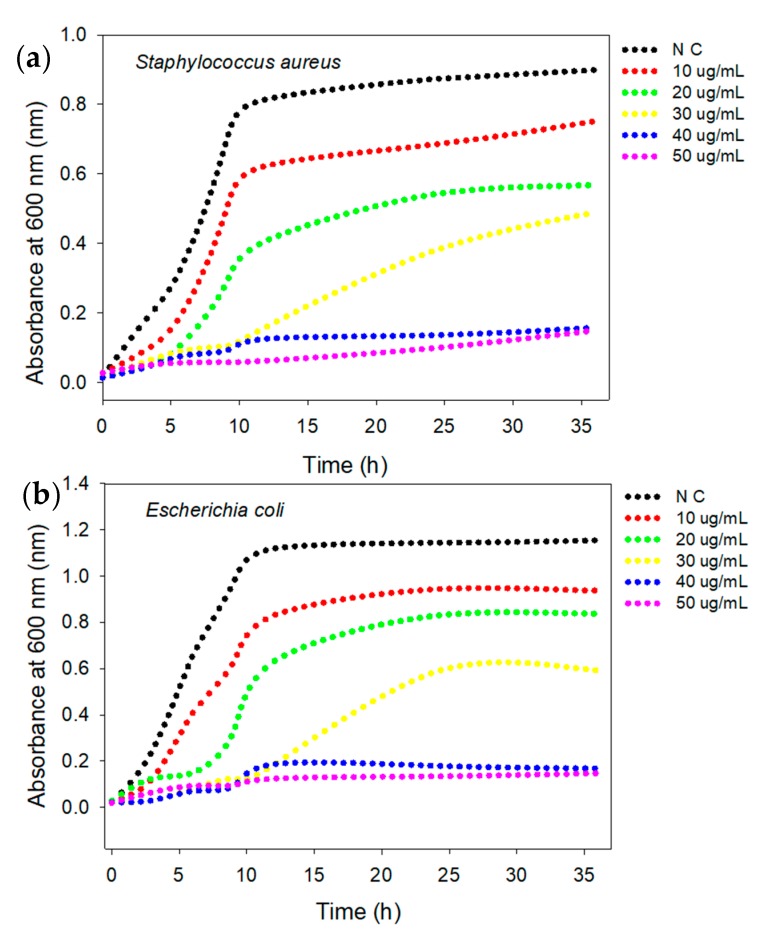
Antibacterial activity of AgNPs against *S. aureus* and *E. coli* using turbidity assay. Bacterial growth curves of *S. aureus* (**a**) and *E. coli* (**b**). Data represent an average of three independent experiments. *N C stands for negative control.

The antibacterial activity of the AgNPs was evaluated against MDR strains of *S. aureus* (KCCM 11335, Gram-positive) and *E. coli* (KCCM 11234, Gram-negative) in LB and NB media using a turbidity assay. The results (Figure 5a,b) indicated that the growth of *S. aureus* bacteria was inhibited by up to 50% in the presence of a relatively low concentration of AgNPs (20 μg/mL). The lag phase of the *S. aureus* bacterial growth curve was extended by up to 12 h compared to the control cells at an AgNP concentration of 30 μg/mL. A further increase in the concentration of the AgNPs to 40 μg/mL caused efficient inhibition of both *S. aureus* and *E. coli* growth, with a respective absorbance of 0.134 and 0.179 at 24 h. Thus, 40 μg/mL was considered the MIC, defined as the minimum concentration of AgNPs that prevents visible growth of both *S. aureus* and *E. coli*. The negative control experiment with cell-free beef extract showed high turbidity, absorbance intensity and bacterial growth. The AgNPs prepared using cell-free beef extract, therefore, showed a concentration-dependent antibacterial activity in preventing the growth of both of the tested bacteria, as suggested in a previous report [46]. It is important to highlight well-established mechanisms in the literature: physical disruption affects normal membrane functioning [47] and the precipitation of bacterial proteins by the release of Ag^+^ ions [48]. The MIC value obtained against clinically known MDR bacteria suggests that AgNPs are inherently effective, with potent antibacterial activity to target the development of resistance in bacteria [49,50].

### 3.7. Bactericidal Concentration

**Figure 6:**
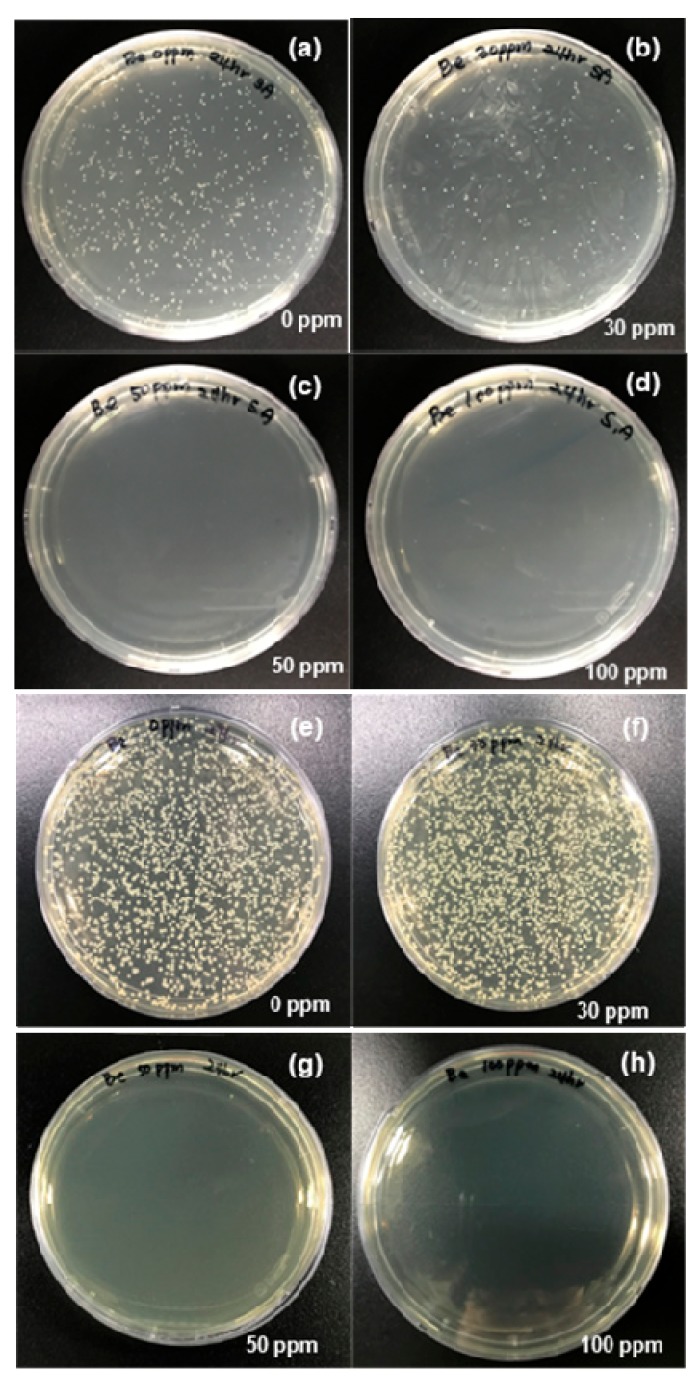
Colony-forming unit (CFU) assays. *S. aureus* cells grown in the absence of AgNPs for 24 h (**a**); *S. aureus* cells grown in the presence of 30 µg/mL AgNPs (**b**); *S. aureus* cells grown in the presence of 50 µg/mL AgNPs (**c**); *S. aureus* cells grown in the presence of 100 µg/mL AgNPs (**d**); *E. coli* cells grown in the absence of AgNPs (**e**); *E. coli* cells grown in the presence of 30 µg/mL AgNPs (**f**); *E. coli* cells grown in the presence of 50 µg/mL AgNPs (**g**); and *E. coli* cells grown in the presence of 100 µg/mL AgNPs (**h**).

*S. aureus* and *E. coli* colony count assays were performed at AgNP concentrations ranging from 0 to 100 µg/mL at 37 °C for 24 h. *S. aureus* colonies were visible on the nutrient agar surface treated with 30 µg/mL AgNPs (Figure 6a,b). However, *S. aureus* cells were eliminated after treatment with 50 µg/mL AgNPs (Figure 6c,d), which demonstrated the potent bacteriostatic activity of the AgNPs. The same method was used to assess the bactericidal activity of the AgNPs against *E. coli* on LB nutrient agar plates. CFUs appeared on both the control plate and the plate treated with 30 µg/mL AgNPs (Figure 6e,f). However, significant bactericidal activity was observed against *E. coli* after treatment with 50 µg/mL AgNPs. This was indicated by a CFU value of nearly zero (Figure 6g,h). Relative to the AgNP control, treatment with 50 µg/mL AgNPs resulted in comparable CFU reductions of 97.5% and 96.7% against MDR *E. coli* and *S. aureus*, respectively. These results showed that the AgNPs prepared in aqueous media had noteworthy activity against both Gram-negative and Gram-positive bacteria. The death of *E. coli* and *S. aureus* occurred immediately after exposure to the AgNPs. In accordance with a previous report, the AgNPs exhibited effective antibacterial activity [51].

### 3.8. Optical Microscopy

**Figure 7:**
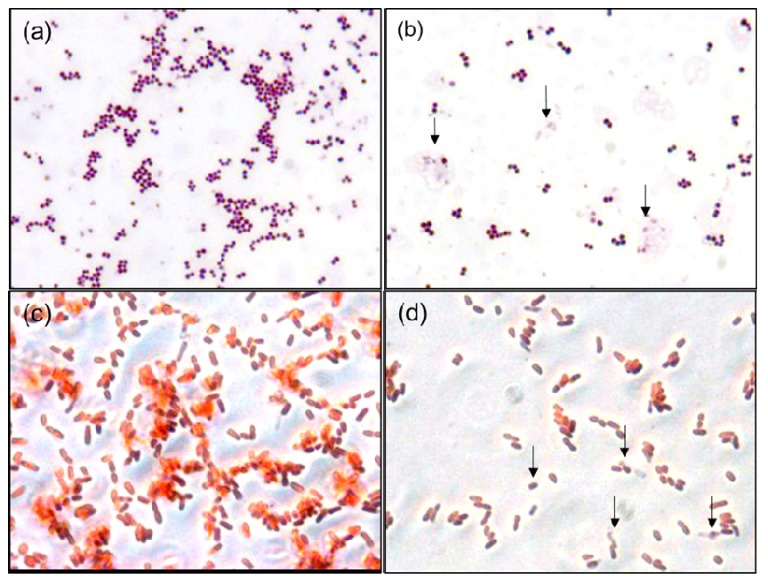
Gram-stained *S. aureus* in the absence of AgNPs (**a**); Gram-stained *S. aureus* in the presence of 30 µg/mL AgNPs (**b**); Gram-stained *E. coli* in the absence of AgNPs (**c**); and Gram-stained *E. coli* in the presence of 30 µg/mL AgNPs (**d**).

Gram staining is a facile and inexpensive laboratory method for the visualization of bacterial morphology and provides useful information for the diagnosis and treatment of bacterial infections. Although AgNPs are well-known antibacterial agents, analysis of their effects on bacterial cell morphology via Gram staining has never been reported. Morphological changes in bacteria in the absence and presence of 30 µg/mL AgNPs were evaluated by the Gram staining of cell samples and examination at 2000x magnification (Figure 7). Compared to control cells, AgNP treatment resulted in diminished interaction between the *S. aureus* cell membrane and the primary stain, resulting in a faint purple color (Figure 7a,b). Disruption of the cell wall and cellular debris was evident in the *S. aureus* sample, indicated by the arrows in Figure 7b. In contrast, the shape, morphology and size of *E. coli* cells remained intact after treatment (Figure 7c,d). It was difficult to discern the morphologies of individual cells in optical microscope images of Gram-stained cells. However, morphological changes were observed in the *S. aureus* cells, indicating that the AgNPs had higher potency against Gram-positive bacteria, in agreement with previous reports [52,53].

### 3.9. Field-Emission Scanning Electron Microscopy

**Figure 8:**
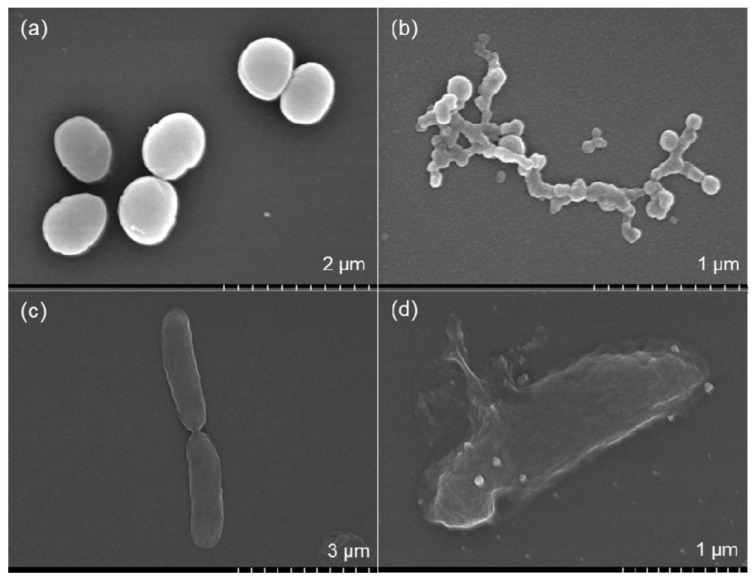
**Field Emission Scanning Electron Microscope (**FE-SEM) image of *S. aureus* in the absence of AgNPs (**a**); FE-SEM image of *S. aureus* in the presence of 30 µg/mL AgNPs (**b**); FE-SEM image of *E. coli* in the absence of AgNPs (**c**); and FE-SEM image of *E. coli* in the presence of 30 µg/mL AgNPs (**d**).

Mechanical damage, altered membrane function and non-viability were observed in the FE-SEM images of *S. aureus* and *E. coli* cells. The results are shown in Figure 8. The membranes of the *S. aureus* cells were completely disrupted and wire-shaped clusters of cellular debris were observed (Figure 8b). A significant number of *S. aureus* cells failed to retain their oval shapes after treatment with 30 µg/mL AgNPs. The formation of residual lipid vesicles and the distortion of cell walls indicated the bactericidal action of the AgNPs. These results suggested that interference with cell membrane permeability affected the viability of the cells. The FE-SEM images also indicated the formation of porous structures on the surfaces of *E. coli* cells after treatment with 30 µg/mL AgNPs (Figure 8d). Cell disruption accompanied by cellular fluid discharge was observed in the *E. coli* cells, which indicated the loss of cell wall integrity. *E. coli* cell disruption and the outflow of cytoplasmic fluid were governed by interactions at the nano-bio interfaces, which caused mechanical damage in the membranes. Interactions between the AgNPs and cell wall components, particularly proteins, may have inhibited the bacterial growth. This phenomenon was reported previously [51]. Crenelated cell surfaces and resultant cellular debris are clear indicators of pronounced antibacterial activity by AgNPs against MDR pathogens [54].

## 4. Conclusions

The biosynthesis of AgNPs was performed with cell-free beef extract using a one-pot, green chemistry approach. The concentrations of NaOH, the reducing agent and AgNO_3_ in this system influenced the evolution of the UV-vis spectra and the yield of colloidal AgNPs. In addition to acting as a reducing reagent and ligand stabilizer, the cell-free extract served as a pH mediator. The prepared AgNPs displayed bacteriostatic activity in a dose-dependent manner and were effective against Gram-negative *E. coli* and Gram-positive *S. aureus*. Optical microscope images confirmed that interactions between the AgNPs and the bacterial cells altered the shape of the colonies. This caused mechanical damage in the cell membranes, altering their surface rigidity, roughness and adhesion potential. The FE-SEM images also suggested that interference with membrane function may have altered the structural and mechanical properties of the membranes, resulting in bacterial non-viability. FE-SEM imaging of individual cells revealed the disruption of cell membranes, the presence of cellular debris and cytoplasmic fluid discharge.
